# Differences in survival of prostate cancer Gleason 8–10 disease and the establishment of a new Gleason survival grading system

**DOI:** 10.1002/cam4.3571

**Published:** 2020-11-01

**Authors:** Yuan Zhou, Changming Lin, Zhihua Hu, Cheng Yang, Rentao Zhang, Yinman Ding, Zhengquan Wang, Sha Tao, Yanmei Qin

**Affiliations:** ^1^ Department of Urology Surgery The People’s Hospital of Xuancheng City Xuancheng China; ^2^ Wannan Medical College Yijiang China; ^3^ Department of Urology Surgery The Fourth Affiliated Hospital of AnHui Medical University Hefei China; ^4^ Department of Urology Surgery The First Affiliated Hospital of Anhui Medical University Hefei China; ^5^ Shanghai Key Laboratory of Tuberculosis Clinic and Research Center of Tuberculosis Shanghai Pulmonary Hospital Tongji University School of Medicine Shanghai China

**Keywords:** Gleason grading system, Gleason score, prostate cancer, SEER, survival

## Abstract

**Background:**

Although the latest Gleason grading system in 2014 has distinguished between Gleason 3 + 4 and 4 + 3, Gleason 8 and Gleason 9–10 are remained systemically classified.

**Methods:**

A total of 261,125 patients diagnosed with prostate cancer (PCa) were selected between 2005 and 2015 from the Surveillance, Epidemiology, and End Results (SEER) database. We used propensity score matching to balance clinical variables and then compared overall survival (OS) and cancer‐specific survival (CSS) between Gleason score subgroups. We further establish a new Gleason survival grading system based on the hazard ratio (HR) values of each Gleason subgroup. Cox proportional hazards models and Kaplan–Meier curves were used to compare patient survival.

**Results:**

Among PCa patients with Gleason score 8 disease, patients with Gleason 5 + 3 had significantly worse OS and CSS than those with Gleason 3 + 5 (OS: HR = 1.26, *p* = 0.042; CSS: HR = 1.42, *p* = 0.005) and 4 + 4 (HR = 1.50 for OS and HR = 1.69 for CSS, *p* < 0.001 for all). PCa patients with Gleason 5 + 3 and Gleason 4 + 5 may have the similar OS and CSS (reference Gleason score <=6, 5 + 3: OS HR = 2.44, CSS HR = 7.63; 4 + 5: OS HR = 2.40, CSS HR = 8.92; *p* < 0.001 for all). The new Gleason survival grading system reclassified the grades 4 and 5 of the 2014 updated Gleason grading system into three hierarchical grades, which makes the classification of grades more detailed and accurate.

**Conclusion:**

PCa patients with Gleason 8–10 may have three different survival subgroups, Gleason 3 + 5 and 4 + 4, Gleason 5 + 3 and 4 + 5, and Gleason 5 + 4 and 5 + 5. Our results maximize risk stratification for PCa patients, provide guidance for clinicians to assess their survival and clinical management, and make a recommendation for the next Gleason grading system update.

## Graphical abstract

We conducted a large‐scale statistical study to analyze the effects of different Gleason scores on overall survival. We further established a new Gleason survival grading system based on the hazard ratio values of each Gleason subgroup.

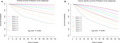

## 1 INTRODUCTION

Prostate cancer (PCa) is the second most common malignancy in men worldwide, which has become the most common malignancy in American men accounting for 28% of all malignancies.^1^, ^2^ Clinically, PCa patients were divided into low‐, medium‐, and high‐risk groups according to their combined prostate‐specific antigen (PSA) values, tumor staging, and Gleason scores, using which can preliminary predict the prognosis of patients and guide treatment decisions.^3^ Gleason scoring system, the pathological grading system for prostate adenocarcinoma, was introduced in 1974 and has become an important predictor of the survival of PCa patients.^4^, ^5^ According to Gleason scoring system, the PCa tissues are divided into primary grading areas and secondary grading areas, each with a score of 1 to 5, and the higher score representing the higher grade of malignancy. However, the 25 different combinations may not be all existent in clinical practice. PCa biopsies with a sum of Gleason score less than 4 (e.g., 1 + 1, 1 + 2, 2 + 1) are almost nonexistent, and the primary and secondary pathologic differences greater than 2 (e.g., 1 + 4, 1 + 5, 4 + 1) are also infrequent.^6^

The Gleason grading system was first codified by International Society of Urological Pathology (ISUP) in 2005, but it contains only the total points to assess the survival of PCa patients.^6^ Many subsequent studies found that PCa patients with Gleason 4 + 3 group had significantly worse prognosis than those with Gleason 3 + 4 group.^7^, ^8^, ^9^ The ISUP updated the latest Gleason grading system in 2014, which further clarified the clinical significance difference between Gleason score 3 + 4 and 4 + 3.^10^ The 2014 ISUP ranged Gleason score into five levels, grade 1: Gleason 2–6; grade 2: Gleason 7 (3 + 4), grade 3: Gleason 7 (4 + 3), grade 4: Gleason 8, and grade 5: Gleason 9–10.^10^ However, the 2014 ISUP Gleason grading system is heterogeneous at grades 4 and 5, Gleason score 8 (grade 4) can be divided into three categories (4 + 4, 3 + 5 and 5 + 3), and Gleason 9–10 (grade 5) can also be divided into three categories (4 + 5, 5 + 4 and 5 + 5), and similar prognosis and treatment were adopted in the same grade.^10^ Is there a significant difference in the survival of these subgroups in the same Gleason grade just like the survival differences between Gleason 3 + 4 and 4 + 3?

To maximize the risk stratification of PCa patients and to enable them to obtain personalized survival prediction and treatment, we conducted a large‐scale statistical study to analyze the effects of different Gleason scores on overall survival (OS) and cancer‐specific survival (CSS) in patients with PCa.

## 2 MATERIALS AND METHODS

### 2.1 DATA acquisition

The patients’ data used for analysis in this study were acquired from Surveillance, Epidemiology, and End Results (SEER) database [SEER 18 Regs Custom Data (with additional treatment fields), November 2018 submission, vision 8.3.5]. The SEER database contains 18 cancer registries which cover about 28% of the United States population.^11^ Patients’ information provided by SEER database has greatly facilitated clinical cancer research.

### 2.2 Study population

Patients diagnosed with PCa between 2005 and 2015 from SEER database were selected. All of the PCa patients had no history of other cancers. We only included patients with pathologically diagnosed prostate adenocarcinoma. Available patient information, including race, age, marital status, TNM stage, PSA value, Gleason score (primary score and secondary score), radiotherapy, surgery, and chemotherapy, were collected. The patients with missing abovementioned information were excluded. PCa patients with scarce Gleason scores, such as Gleason 2 + 4, 3 + 1 and 4 + 2, were not included in our study.

### 2.3 Statistical analysis

To analyze the effect of Gleason score on survival in PCa patients as accurately as possible, we used propensity score matching (PSM) to balance clinical variables and minimize statistical bias, and then compare patient survival. The patient information, race, age, marital status, TNM stage, PSA value, radiotherapy, surgery, and chemotherapy, were matched between PCa patients with Gleason score less than or equal to 6 and other Gleason score values. We further compared the OS and CSS for each patient characteristic subgroup between PCa patients with Gleason score less than or equal to 6 and other Gleason score values after PSM adjusted. We pairwise compared the overall death risk and cancer‐specific death risk between subgroups of PCa patients with Gleason 7, 8, and 9–10 after PSM adjusted. Cox proportional hazards model was used to compare overall and cancer‐specific death risk of patients. We further establish a new Gleason survival grading system of PCa based on the hazard ratio (HR) values of each Gleason subgroup after PSM adjusted. We used Kaplan–Meier curves to compare the OS and CSS of PCa patients divided with different grades using the new Gleason survival grading system and with the 2014 ISUP Gleason grading system.

Overall death and cancer‐specific death were considered as the primary endpoints of this study. In the OS analysis, alive patients were considered as censored data. In the CSS analysis, alive patients and patients who died not due to the cancer are considered as censored data. All statistical analyses were implement by R software 3.6.2, and two‐sided *p* values less than 0.05 were determined as statistical significance.

## 3 RESULTS

### 3.1 Baseline patient characteristics

A total of 261,125 patients diagnosed with PCa between 2005 and 2015 were included in this study. Gleason scores for PCa patients contain less than or equal to 6 (2 + 3, 3 + 2, 3 + 3), 3 + 4, 3 + 5, 4 + 3, 4 + 4, 4 + 5, 5 + 3, 5 + 4, and 5 + 5. Baseline characteristics including race, age, marital status, TNM stage, PSA value, radiotherapy, surgery, and chemotherapy of each Gleason subgroup are listed in Table 1. The distribution of invasion factors of PCa, TNM stage, and PSA values is shown in Table 2. Notably, we found that PCa patients with higher Gleason score, T stage, and PSA value distribution were more likely to have regional lymph node and distant metastasis (Table 2).

**TABLE 1:** Baseline characteristics of prostate cancer patients from SEER database (*n* = 261,125, distribution difference *p* < 0.001 for all)

Patient characteristics	Gleason score (Number of patients)								
	<=6	3 + 4	3 + 5	4 + 3	4 + 4	4 + 5	5 + 3	5 + 4	5 + 5
Race									
White	90,425	59,091	1,789	24,249	14,762	9,906	509	2,988	1,472
Black	16,963	12,890	508	5,410	3,320	2,007	130	637	304
Other	5,442	3,644	122	1,865	1,413	873	56	304	146
Age									
<=50	6,713	3,753	83	953	435	359	17	103	51
51–60	34,939	22,107	650	7,495	3,594	2,501	137	710	326
61–70	49,058	33,510	1,082	1,4058	8,084	5,141	289	1,529	668
71–80	19,763	14,375	502	7,709	5,911	3,562	197	1,114	531
>80	2,357	1,880	102	1,309	1,471	1,223	55	473	346
Marital status									
Married	87,121	57,581	1,733	23,810	14,563	9,185	501	2,790	1,276
Divorced	8,430	5,931	228	2,581	1,463	1,113	54	368	188
Separated	991	768	46	286	192	158	7	46	24
Widowed	3,766	2,678	124	1,352	1,040	768	42	248	151
Single	12,522	8,667	288	3,495	2,237	1,562	91	477	283
T (tumor invasion)									
T1a	3,426	449	11	107	63	27	4	10	12
T1b	916	537	33	249	176	293	17	165	79
T1c	57,522	25,374	730	11,228	7,661	4,189	229	1,261	646
T2a	11,438	6,104	121	2,352	1,043	544	34	131	46
T2b	1,793	2,908	90	1,201	876	506	21	145	49
T2c	33,075	29,023	640	8,709	4,234	2,157	149	611	275
T3a	3,349	8,428	458	4,617	2,537	1,948	117	492	154
T3b	714	2,673	247	2,407	1,802	2,134	88	635	249
T4	597	939	89	654	748	988	36	479	412
N (regional lymph node)									
No	112,597	74,700	2,260	30,382	1,8044	10,817	639	3,197	1,420
Yes	233	925	159	1,142	1,451	1,969	56	732	502
M (metastasis)									
No	112,585	75,139	2,315	30,882	1,8096	10,888	632	3,071	1,290
Yes	245	486	104	642	1,399	1,898	63	858	632
PSA value									
<=20	4,917	1,697	22	497	310	180	10	75	45
21–40	14,595	7,658	145	2,174	1,062	640	27	190	67
41–60	42,625	26,520	602	8,622	4,068	1,862	142	490	196
61–80	21,697	15,344	431	6,103	3,186	1,755	114	454	183
81–100	10,429	7,906	288	3,735	2,110	1,273	66	330	113
>100	18,567	16,500	931	10,393	8,759	7,076	336	2,390	1,318
Surgery									
No surgery	61,476	30,247	991	14,509	10,922	6,984	337	2,262	1,248
Radical prostatectomy	44,414	42,631	1,315	15,806	7,643	4,853	310	1,199	331
Other surgical methods	6,940	2,747	113	1,209	930	949	48	468	343
Radiation									
Beam radiation	19,715	17,707	705	9,638	7,429	4,958	251	1,555	776
Combination beam with implants/isotopes	2,803	3,693	111	2,015	1,451	679	33	170	58
Radioactive implants	14,942	4,461	52	1,370	530	216	22	41	21
Refused	704	371	20	199	107	98	3	36	9
None/Unknown	74,666	49,393	1,531	18,302	9,978	6,835	386	2,127	1,058
Chemotherapy									
Yes	122	158	29	161	264	405	13	3,751	115
None/Unknown	112,708	75,467	2,390	31,363	19,231	12,381	682	178	1,767
Death status									
Alive	100,982	67,218	1,912	27,001	15,524	8,958	461	2,300	914
Death	11,848	8,407	507	4,523	3,971	3,828	234	1,629	1,008
Cause of death									
By cancer	1,301	1,443	189	1,236	1,649	2,276	107	1,112	745
Other cause or alive	111,529	74,182	2,230	30,288	17,846	10,510	588	2,817	1,177
Total	112,830	75,625	2,419	31,524	19,495	12,786	695	3,929	1,922

**TABLE 2:** The distribution of invasion factors of prostate cancer (n = 261,125)

Patient characteristics	Gleason score (Number of patients)								
	<=6	3 + 4	3 + 5	4 + 3	4 + 4	4 + 5	5 + 3	5 + 4	5 + 5
Number of patients	112,830	75,625	2,419	31,524	19,495	12,786	695	3,929	1,922
T stage (%)									
T1	54.8	34.9	32.0	36.7	40.5	35.3	35.9	36.5	38.3
T2	41.0	49,2	35.2	38.9	33.4	25.1	29.4	22.6	19.3
T3	3.6	14.7	29.1	22.2	22.2	31.9	29.5	28.7	21.0
T4	0.5	1.3	3.7	2.1	3.8	7.7	5.2	12.2	21.4
N stage (%)									
No	99.8	98.8	93.4	96.4	92.6	84.6	91.9	81.4	73.9
Yes	0.2	1.2	6.6	3.6	7.4	15.4	8.1	18.6	26.1
M stage (%)									
No	99.8	99.4	95.7	98.0	92.8	85.2	90.9	78.2	67.1
Yes	0.2	0.6	4.3	2.0	7.2	14.8	9.1	21.8	32.9
PSA value (ng/ml)									
Median	57	62	81	74	91	116	95	139	201.5
IQR	44–82	47–93	56–158	53–123	58–190	66–321	66–212.5	70–465	80–860

Percentages may not add up to 100 due to rounding.

Abbreviations: IQR, interquartile range.

### 3.2 Association between Gleason score and survival

**FIGURE 1:**
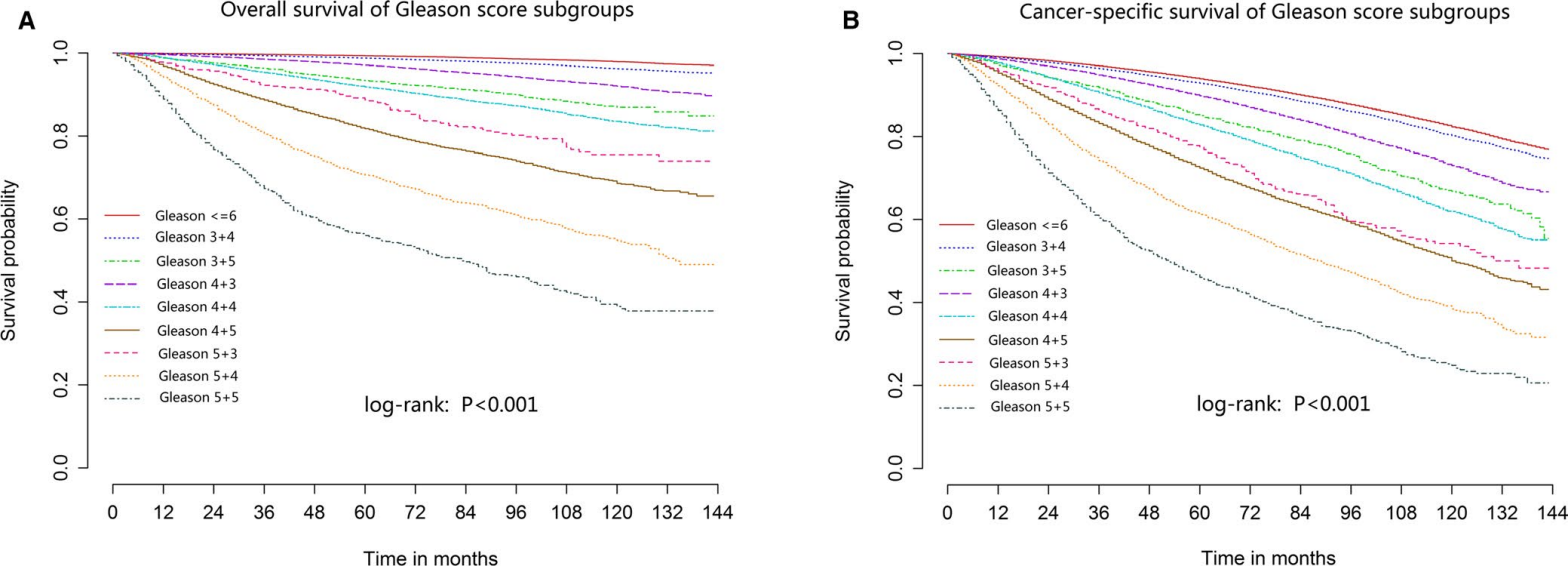
Kaplan–Meier curves are shown for the comparison of overall survival (A) and cancer‐specific survival (B) between prostate cancer patients with different Gleason scores values

Kaplan–Meier curve was used to preliminary compare the OS and CSS of PCa patients with different Gleason scores values (Figure 1). We further used PSM to balance clinical variables and minimize statistical bias, and then compare the OS and CSS between PCa patients with Gleason score less than or equal to 6 and other Gleason subgroups. The comparisons of overall death risk and all cancer‐specific death risk between Gleason subgroups are listed in Table 3, we found in PCa patients with Gleason score of 8, patients with Gleason 5 + 3 may have significantly worse OS and CSS than those with 3 + 5 and 4 + 4 (reference Gleason score <=6, 5 + 3: OS HR = 2.44, 95%CI = 2.08–2.85, CSS HR = 7.63, 95%CI = 5.77–10.01; 3 + 5: OS HR = 2.08, 95%CI = 1.87–2.30, CSS HR = 5.15, 95%CI = 4.21–6.30; 4 + 4: OS HR = 1.62, 95%CI = 1.55–1.70, CSS HR = 4.06, 95%CI = 3.68–4.48; *p* < 0.001 for all). Among PCa patients with Gleason score 9–10 disease, the OS and CSS of patients with Gleason 4 + 5 may have significantly better than those with 5 + 4 and 5 + 5 (reference Gleason score <=6, 4 + 5: OS HR = 2.40, 95%CI = 2.28–2.53, CSS HR = 8.92, 95%CI = 8.05–9.87; 5 + 4: OS HR = 3.34, 95%CI = 3.09–3.60, CSS HR = 14.76, 95%CI = 12.95–16.83; 5 + 5: OS HR = 4.00, 95%CI = 3.61–4.44, CSS HR = 17.62, 95%CI = 14.84–20.92; *p* < 0.001 for all, Table 3). We further divided patients into subgroups based on the characteristics of the patients, and compared the OS and CSS among patients with different Gleason score in each subgroup after PSM adjusted and found that in almost all subgroups, patients with Gleason 8–10 may have three different survival subgroups: Gleason 3 + 5 and 4 + 4, Gleason 5 + 3 and 4 + 5, and Gleason 5 + 4 and 5 + 5 (Tables 4 and 5). We pairwise compared the overall death risk and cancer‐specific death risk between subgroups of PCa patients with Gleason 7, 8, and 9–10 after PSM adjusted, and found that patients with Gleason 5 + 3 did have higher overall death and cancer‐specific death than patients with Gleason 3 + 5 (OS: HR = 1.26, *p* = 0.042; CSS: HR = 1.42, *p* = 0.005) and 4 + 4 (HR = 1.50 for OS and HR = 1.69 for CSS, *p* < 0.001 for all) disease. In three subgroups of Gleason score 9–10, Gleason 5 + 4 and 5 + 5 increased 0.31‐ and 0.76‐fold for overall death risk and 0.51‐ and 1.02‐fold for cancer‐specific death risk (Table 6). The comparison of survival between Gleason 5 + 3 and Gleason 4 + 5 was not statistically significant even after PSM adjusted (*p* = 0.684 for OS and *p* = 0.091 for CSS).

**TABLE 3:** The comparison of overall death risk and all cancer‐specific death risk between prostate cancer patients with Gleason score less than or equal to 6 and other Gleason score values

Gleason score	Overall HR (95% CI)	*P* value	Cancer‐specific HR (95% CI)	*P* value
<=6	Reference		Reference	
3 + 4	1.21 (1.17–1.24)	<0.001	1.60 (1.47–1.74)	<0.001
4 + 3	1.39 (1.33–1.44)	<0.001	2.59 (2.35–2.85)	<0.001
3 + 5	2.08 (1.87–2.30)	<0.001	5.15 (4.21–6.30)	<0.001
4 + 4	1.62 (1.55–1.70)	<0.001	4.06 (3.68–4.48)	<0.001
5 + 3	2.44 (2.08–2.85)	<0.001	7.63 (5.77–10.01)	<0.001
4 + 5	2.40 (2.28–2.53)	<0.001	8.92 (8.05–9.87)	<0.001
5 + 4	3.34 (3.09–3.60)	<0.001	14.76 (12.95–16.83)	<0.001
5 + 5	4.00 (3.61–4.44)	<0.001	17.62 (14.84–20.92)	<0.001

All comparisons were performed after propensity matching score adjusted.

Abbreviations: CI, confidence interval; HR, hazard ratio.

**TABLE 4:** Multivariable covariate‐adjusted hazard ratio (HR) for overall death risk between prostate cancer patients with Gleason score less than or equal to 6 and other Gleason score values

Patient characteristics	Gleason Score															
	3 + 4		3 + 5		4 + 3		4 + 4		4 + 5		5 + 3		5 + 4		5 + 5	
	HR	*P*	HR	*P*	HR	*P*	HR	*P*	HR	*P*	HR	*P*	HR	*P*	HR	*P*
Reference Gleason<=6																
Race																
White	1.19	※	2.05	※	1.43	※	1.75	※	2.65	※	2.60	※	3.71	※	4.89	※
Black	1.13	※	1.79	※	1.28	※	1.50	※	2.20	※	2.41	※	3.16	※	3.29	※
Other	1.18	.029	2.02	.004	1.41	※	1.70	※	2.19	※	1.73	.155	3.67	※	3.64	※
Age																
<=60	1.38	※	2.77	※	1.68	※	1.99	※	3.75	※	3.69	※	5.42	※	7.24	※
61–70	1.22	※	2.19	※	1.48	※	1.78	※	2.77	※	2.49	※	3.99	※	5.09	※
>70	1.13	※	1.64	※	1.29	※	1.58	※	2.16	※	2.15	※	2.97	※	3.66	※
Marital Status																
Married	1.17	※	2.13	※	1.41	※	1.73	※	2.61	※	2.81	※	3.69	※	3.77	※
Di/Se/Wi	1.18	※	1.71	※	1.37	※	1.61	※	2.06	※	2.10	※	3.13	※	4.02	※
Single	1.28	※	1.94	※	1.32	※	1.60	※	2.95	※	2.22	※	4.16	※	5.96	※
T Stage																
T1	1.29	※	1.87	※	1.40	※	1.69	※	2.41	※	2.26	※	3.20	※	4.06	※
T2	1.12	※	1.94	※	1.37	※	1.50	※	2.15	※	1.72	※	3.09	※	4.17	※
T3/T4	1.20	.011	2.00	※	1.39	※	1.69	※	2.61	※	2.63	※	3.54	※	4.22	※
N Stage																
N0	1.19	※	2.02	※	1.40	※	1.70	※	2.55	※	2.61	※	3.61	※	4.58	※
N1	1.40	.156	1.90	.093	1.58	.055	1.22	.350	1.94	※	2.09	.137	3.01	※	2.45	※
M Stage																
M0	1.19	※	2.02	※	1.41	※	1.72	※	2.59	※	2.51	※	3.75	※	4.71	※
M1	1.28	.082	1.65	.027	1.22	.146	1.30	.031	1.80	※	2.59	※	2.09	※	2.38	※
PSA value																
<=40	1.13	.001	2.20	※	1.31	※	1.69	※	2.78	※	4.96	※	5.32	※	3.72	※
40–100	1.18	※	1.98	※	1.45	※	1.68	※	2.40	※	2.63	※	3.47	※	4.65	※
>100	1.25	※	2.04	※	1.35	※	1.65	※	2.50	※	2.47	※	3.29	※	3.97	※
Surgery																
No surgery	1.30	※	1.81	※	1.42	※	1.64	※	2.18	※	1.91	※	2.83	※	3.51	※
Prostatectomy	1.13	※	1.95	※	1.35	※	1.87	※	2.34	※	2.31	※	3.36	※	3.87	※
Other methods	1.12	.016	1.99	※	1.23	※	1.61	※	2.87	※	2.74	※	3.94	※	4.84	※
Radiation																
Yes	1.22	※	1.63	※	1.32	※	1.59	※	1.95	※	2.04	※	2.78	※	3.11	※
None/Unknown	1.22	※	2.28	※	1.51	※	1.90	※	3.09	※	2.86	※	3.99	※	5.23	※
Chemotherapy																
None/Unknown	1.19	※	2.03	※	1.41	※	1.70	※	2.96	※	2.59	※	3.20	※	4.53	※

All comparisons were performed after propensity matching score adjusted.

※ represents the *P* value less than 0.001.

Abbreviations: Di/Se/Wi, Divorced, Separated and Widowed.

Due to the limitation of the number of patients undergoing chemotherapy, the analysis was not performed.

**TABLE 5:** Multivariable covariate‐adjusted hazard ratio (HR) for cancer‐specific death risk between prostate cancer patients with Gleason score less than or equal to 6 and other Gleason score values

Patient characteristics	Gleason score															
	3 + 4		3 + 5		4 + 3		4 + 4		4 + 5		5 + 3		5 + 4		5 + 5	
	HR	*P*	HR	*P*	HR	*P*	HR	*P*	HR	*P*	HR	*P*	HR	*P*	HR	*P*
Reference Gleason<=6																
Race																
White	1.66	※	4.71	※	2.72	※	3.16	※	9.57	※	6.84	※	15.95	※	21.19	※
Black	1.31	.004	4.20	※	1.96	※	3.68	※	7.70	※	8.11	※	8.66	※	10.98	※
Other	1.87	.003	8.12	※	3.09	※	4.72	※	8.10	※	10.04	※	19.49	※	16.27	※
Age																
<=60	1.99	※	7.70	※	3.93	※	6.30	※	18.33	※	15.14	※	25.92	※	31.21	※
61–70	1.57	※	4.65	※	2.92	※	4.41	※	9.35	※	7.92	※	16.95	※	22.66	※
>70	1.57	※	3.70	※	1.99	※	3.42	※	6.51	※	4.35	※	11.04	※	12.61	※
Marital status																
Married	1.55	※	5.30	※	2.57	※	4.14	※	9.50	※	7.87	※	11.62	※	16.37	※
Di/Se/Wi	1.52	※	2.97	※	2.52	※	3.76	※	6.47	※	5.69	※	8.24	※	12.66	※
Single	2.02	※	6.18	※	2.75	※	4.65	※	11.55	※	7.09	※	11.12	※	28.99	※
T Stage																
T1	1.77	※	4.27	※	2.49	※	3.93	※	8.51	※	5.48	※	13.36	※	16.27	※
T2	1.57	※	3.09	※	2.73	※	4.08	※	7.90	※	4.22	※	12.66	※	19.45	※
T3/T4	1.67	.001	5.72	※	2.84	※	4.03	※	8.08	※	10.35	※	13.70	※	14.32	※
N Stage																
N0	1.60	※	4.68	※	2.57	※	4.10	※	9.16	※	7.28	※	14.98	※	18.25	※
N1	2.11	.037	5.27	.007	2.14	.025	1.87	.028	2.75	※	2.85	.103	5.34	※	4.29	※
M Stage																
M0	1.62	※	5.08	※	2.67	※	4.44	※	10.08	※	7.85	※	17.81	※	23.15	※
M1	1.51	.211	2.41	.001	1.48	.020	1.63	※	2.33	※	2.96	※	2.87	※	3.13	※
PSA value																
<=40	1.36	.044	8.50	※	3.66	※	5.20	※	16.60	※	20.20	※	34.78	※	33.82	※
40–100	1.59	※	4.83	※	2.75	※	4.02	※	8.80	※	9.13	※	17.47	※	25.92	※
>100	1.69	※	4.57	※	2.30	※	3.84	※	7.79	※	5.68	※	11.14	※	13.58	※
Surgery																
No surgery	1.73	※	3.50	※	2.48	※	3.66	※	6.86	※	5.83	※	10.55	※	12.55	※
Prostatectomy	1.62	※	5.17	※	3.18	※	5.36	※	11.78	※	11.02	※	21.10	※	22.23	※
Other methods	2.10	※	5.77	※	2.79	※	5.67	※	15.01	※	10.17	※	20.10	※	24.60	※
Radiation																
Yes	1.54	※	3.20	※	2.32	※	3.31	※	6.20	※	5.19	※	11.51	※	12.59	※
None/Unknown	1.76	※	5.69	※	2.85	※	4.93	※	11.78	※	7.66	※	16.53	※	19.60	※
Chemotherapy																
None/Unknown	1.60	※	4.85	※	2.61	※	4.17	※	9.25	※	7.18	※	15.27	※	18.07	※

All comparisons were performed after propensity matching score adjusted.

※ represents the *P* value less than 0.001.

Abbreviations: Di/Se/Wi, Divorced, Separated and Widowed.

Due to the limitation of the number of patients undergoing chemotherapy, the analysis was not performed.

**TABLE 6:** The comparison of overall death risk and cancer‐specific death risk between subgroups of prostate cancer patients with Gleason 7, 8, and 9–10

Gleason score	Overall HR (95% CI)	*P* value	Cancer‐specific HR (95% CI)	*P* value
4 + 3 vs. 3 + 4	1.15 (1.11–1.20)	<0.001	1.60 (1.47–1.75)	<0.001
3 + 5 vs. 4 + 3	1.45 (1.30–1.61)	<0.001	1.72 (1.42–2.08)	<0.001
5 + 3 vs. 3 + 5	1.26 (1.01–1.58)	0.042	1.42 (1.11–1.81)	0.005
5 + 3 vs. 4 + 4	1.50 (1.29–1.75)	<0.001	1.69 (1.33–2.15)	<0.001
3 + 5 vs. 4 + 4	1.13 (1.02–1.25)	0.016	1.10 (0.91–1.33)	0.315
4 + 5 vs. 5 + 3	1.03 (0.89–1.19)	0.684	1.19 (0.97–1.47)	0.091
5 + 4 vs. 4 + 5	1.31 (1.22–1.41)	<0.001	1.51 (1.38–1.65)	<0.001
5 + 5 vs. 4 + 5	1.76 (1.61–1.93)	<0.001	2.02 (1.81–2.54)	<0.001
5 + 5 vs. 5 + 4	1.32 (1.18–1.47)	<0.001	1.40 (1.22–1.60)	<0.001

All comparisons were performed after propensity matching score adjusted.

Abbreviations: CI, confidence interval.

### 3.3 Establishment of new Gleason survival grading system

**FIGURE 2:**
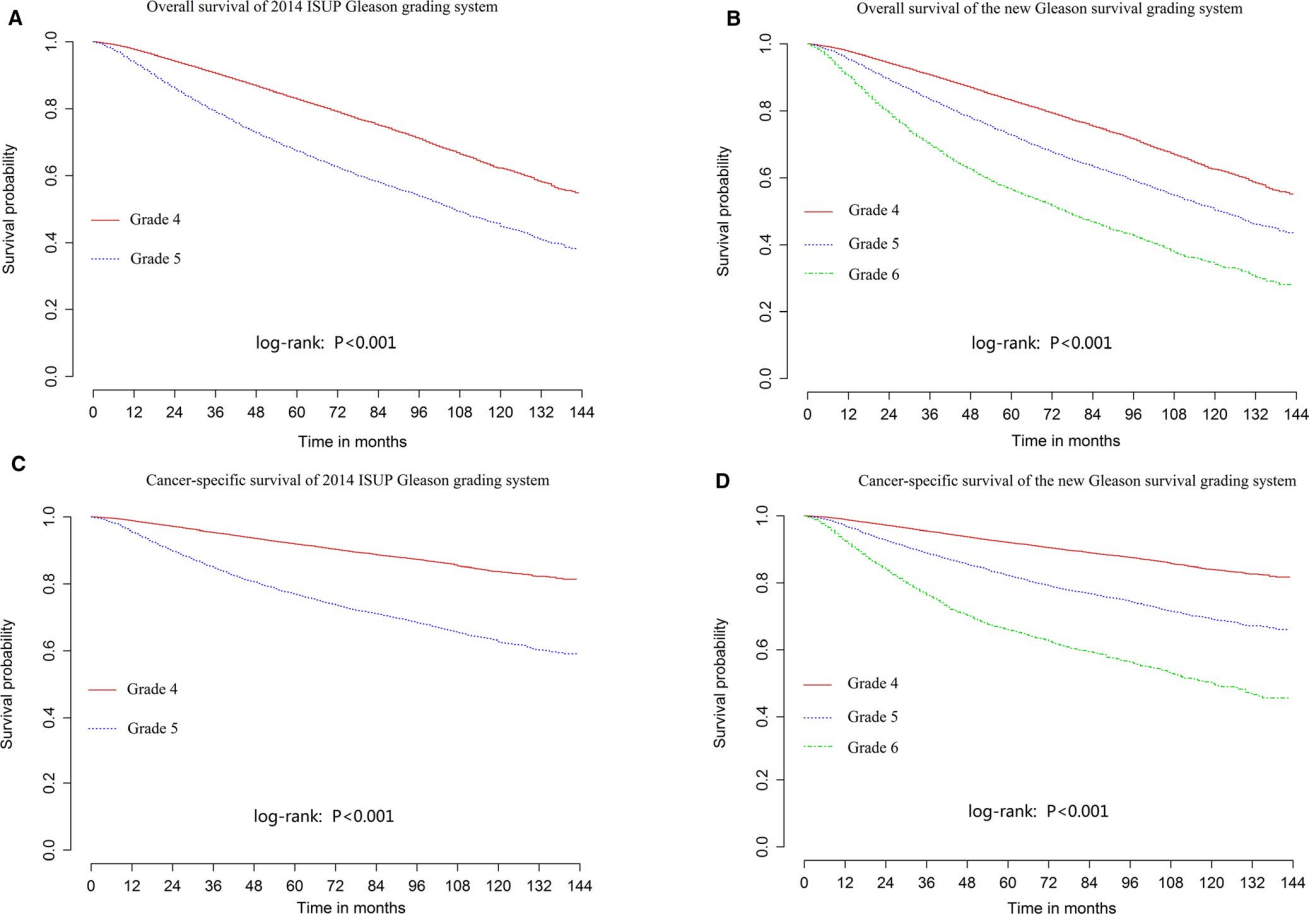
The comparison of overall survival (OS) and cancer‐specific survival (CSS) between prostate cancer patients with different Gleason grades using the new Gleason survival grading system and the 2014 ISUP Gleason grading system. Kaplan–Meier curves for comparison of OS in (A) 2014 ISUP Gleason grading system and (B) the new Gleason survival grading system and CSS in (C) 2014 ISUP Gleason grading system and (D) the new Gleason survival grading system

According to the HRs of the subgroups shown in Tables 3 and 6, we established a new Gleason survival grading system to maximize the risk stratification of PCa patients. The new Gleason survival grading system contains six grades, among which grades 4 to 6 are changed from grade 4 (Gleason 8) and grade 5 (Gleason 9–10) defined by the 2014 ISUP Gleason grading system (Table 7). Kaplan–Meier curves were used to compare the OS and CSS of patients divided with different Gleason grades using the new Gleason survival grading system and the 2014 ISUP Gleason grading system (Figure 2) and we found that the survival of patients with different Gleason grades was more distinct and accurate when our Gleason grading system was used (Table 7).

**TABLE 7:** The comparison of overall survival and cancer‐specific survival rates of prostate cancer patients between 2014 ISUP grading system and the new Gleason survival grading system

Grade	2014 ISUP Gleason grading system			The New Gleason survival grading system		
	Gleason score	5‐year survival rate (%)		Gleason score	5‐year survival rate (%)	
		OS	CSS		OS	CSS
Grade 1	2–6	93.9	99.3	2–6	93.9	99.3
Grade 2	3 + 4	92.8	98.7	3 + 4	92.8	98.7
Grade 3	4 + 3	89.8	97.1	4 + 3	89.8	97.1
Grade 4	8 (4 + 4/3 + 5/5 + 3)	82.9	91.9	4 + 4/3 + 5	83.1	92.0
Grade 5	9–10 (4 + 5/5 + 4/5 + 5)	67.3	76.8	5 + 3/4 + 5	72.3	82.2
Grade 6	None			5 + 4/5 + 5	56.3	65.9

Grades 1 to 3 are the same in both Gleason grading systems.

## 4 DISCUSSION

In 2014, the ISUP developed the latest Gleason grading system after the 2005 version. The modified Gleason grading system was grouped according to the histological characteristics of the Gleason score and the prognosis of the PCa patients, showing a significant improvement over the previous Gleason scoring system: 1) provided a more accurate stratification depending on the pathology of PCa; 2) classified simplifies the division of Gleason scores; and 3) grouped according to the survival of PCa patients.^10^, ^12^ However, the 2014 Gleason grading system still systematically classifies Gleason 8 and Gleason 9–10. Therefore, the purpose of this study was to study in detail the effects of Gleason score values on OS and CSS of PCa patients.

PCa with Gleason score less than or equal to 6 had been considered indolent and had a high cure rate even including with extra‐prostatic invasion and positive margins.^12^, ^13^ Therefore, PCa with Gleason score less than or equal to 6 was set as the matched group in this study. After matching the clinical information of PCa patients, we found that in three subgroups with a Gleason score of 8, the risk of overall death and cancer‐specific death in patients with Gleason 5 + 3 was significantly higher than those with Gleason 3 + 5 and 4 + 4, and in three subgroups of Gleason score 9–10, patients with Gleason 4 + 5 had better OS and CSS than Gleason 5 + 4 and 4 + 5 groups. The comparison of survival between Gleason 5 + 3 and Gleason 4 + 5 was not statistically significant, but they may have the similar OS and CSS (reference Gleason score <=6, 5 + 3: OS HR = 2.44, CSS HR = 7.63; 4 + 5: OS HR = 2.40, CSS HR = 8.92; *p* < 0.001 for all). Currently, the Gleason score of 8–10 is classified as a high‐risk clinical group and all treated with the same clinical guidelines.^3^ Clinicians generally recognized that patients with Gleason 9–10 have a worse prognosis than those with Gleason score of 8, and the difference in prognosis may be one of the factors guiding treatment decisions.^14^ For example, the National Comprehensive Cancer Network (NCCN) guidelines recommend that PCa patients with Gleason 8–10 should receive 2 to 3 years of androgen deprivation therapy before radiation, and some clinicians may be inclined to 2 years for Gleason 8 and 3 years for Gleason 9–10.^15^, ^16^ However, our study showed that PCa patients with Gleason 8–10 had significant differences in OS and CSS in subgroups, which may be divided into three different prognostic subgroups, Gleason 3 + 5 and 4 + 4, Gleason 5 + 3 and 4 + 5, and Gleason 5 + 4 and 5 + 5. Patients in different prognostic groups may require more personalized treatment. We found that the PCa patients with Gleason 3 + 5 had worse OS than whose with Gleason 4 + 4 (HR = 1.13, 95%CI = 1.02–1.25, *p* = 0.016), while the comparison of CSS was significant (HR = 1.10, *p* = 0.315). Previous studies have shown that PCa with Gleason pattern 5 had poorer survival and higher biochemical recurrence rate than patients without Gleason pattern 5.^17^, ^18^ However, due to the influence of primary and secondary pathology, the survival difference between Gleason 4 + 4 and 3 + 5 patients may not be very significant.^19^ Another study reported that PCa patients with Gleason 5 + 3 had a significantly worse CSS than whose with Gleason 4 + 4 or 3 + 5, but similar to Gleason 9 disease, which was basically consistent with our results.^20^ The results of our study might have guiding significance for the development of treatment and follow‐up strategies for PCa patients. We further developed a new Gleason survival grading system based on the survival of PCa patients in each Gleason subgroups. The proposed Gleason survival grading system reclassified the grades 4 and 5 of the 2014 ISUP Gleason grading system into three hierarchical grades, which makes the Gleason grading system more detailed and accurate.

Interestingly, we found that PCa patients with higher Gleason scores had higher T staging and PSA distribution, and were more likely to have regional lymph nodes and distant metastasis. Although PSA has a high proportion of deviations in clinical diagnosis, it is still an important factor affecting the survival of PCA patients and a driving factor for almost all initial disease treatment and management decisions.^3^, ^21^ Studies have shown that PCa patients with higher PSA and Gleason score had a higher risk of developing lymph nodes and distant metastases.^22^, ^23^ Therefore, PCa patients with higher Gleason scores should need more aggressive treatment and closer follow‐up.

Because PCa patients with a Gleason score of 7 are more common than those with a Gleason score of 8–10 (41.0% vs. 15.7% in this study), most studies were focused on the difference in survival between Gleason 3 + 4 and 4 + 3 disease,^7^, ^24^, ^25^ and data on Gleason 8–10 have rarely been collected to study the subgroup survival differences. This study is the first to study the survival differences of Gleason 8–10 subgroups in detail based on large sample size and long‐term follow‐up data of PCa patients, and we hope that our study results can provide a proposal for the next Gleason score update.

Our study has important clinical significance for guiding and changing the clinical nomogram, follow‐up adjustment, and treatment of high‐risk PCa, but it still has some potential limitations. First, the time of biochemical recurrence and PSA growth rate after treatment are important factors affecting the survival of PCa patients,^26^ and the absence of the above information in the SEER database may affect our results. Second, the grade of Gleason score is divided by pathomorphism and the survival of patients, and the proposed new Gleason survival grading system is based entirely on survival of PCa patients. Lastly, our study was retrospective in nature, so more prospective studies are required to verify our results.

## 5 CONCLUSIONS

In conclusion, based on the large sample size and long‐term patient data, we used the statistical method of low inter‐group bias to study the effect of Gleason score on OS and CSS of PCa patients and proposed a new Gleason survival grading system for the first time. Our results provide valid epidemiological evidence that PCa patients with Gleason 8–10 may have three different survival groups, Gleason 3 + 5 and 4 + 4, Gleason 5 + 3 and 4 + 5, and Gleason 5 + 4 and 5 + 5. Our results may provide guidance for clinicians in evaluating survival and clinical management for PCa patients, and provide a suggestion for ISUP to update Gleason score next time.

## CONFLICT OF INTEREST

The authors have no conflict of interest.

## AUTHOR CONTRIBUTIONS

Conceptualization: Yanmei Qin and Yuan Zhou. Data curation: Changming Lin and Zhihua Hu. Formal analysis: Yuan Zhou, Cheng Yang, and Rentao Zhang. Funding acquisition: Zhihua Hu. Methodology: Yuan Zhou and Yinman Ding. Supervision: Yanmei Qin and Zhengquan Wang. Validation: Changming Lin and Sha Tao. Writing – original draft: Yuan Zhou and Zhihua Hu. Writing – review & editing: Yanmei Qin and Changming Lin.

## 6 ETHICS STATEMENT

All patients’ information in the present study were acquired from the SEER database for research purposes. Thus, no human or animal participants were involved in this study.

## Data Availability

All patients’ information in the present study were acquired from the SEER database for research purposes.
